# Cervical Spondylotic Myelopathy Secondary to Dropped Head Syndrome: Report of a Case and Review of the Literature

**DOI:** 10.1155/2016/5247102

**Published:** 2016-03-13

**Authors:** Abolfazl Rahimizadeh, Housain F. Soufiani, Saghayegh Rahimizadeh

**Affiliations:** ^1^Department of Spinal Surgery, Pars Advanced and Minimally Invasive Manners Research Center (PAMIM), Affiliated to Iran University of Medical Sciences, Pars Hospital, Tehran, Iran; ^2^Atlantic University, School of Medicine, P.O. Box 456, Island Park, NY 11558, USA

## Abstract

The dropped head syndrome (DHS) is a disabling condition caused by severe weakness of the neck extensor muscles causing progressive reducible kyphosis of the cervical spine and the inability to hold the head up. Weakness can occur in isolation or in association with a generalized neuromuscular disorder. Isolated cases are owed to the late onset of noninflammatory myopathy designated as INEM, where persistent chin to chest deformity may gradually cause or aggravate preexisting degenerative changes of the cervical spine and ultimately result in myelopathy. In review of the literature, we could find only 5 cases, with no unique guidelines to address the management of these two concomitant pathologies. Herein, a 69-year-old man who had developed cervical myelopathy 2 years after being affected by isolated dropped head syndrome is presented. Chin to chest deformity and cervical myelopathy were managed through three-level anterior cervical discectomy and fusion (ACDF) combined with decompressive cervical laminectomy and stabilization with C2 to C7 pedicle screw-rod construct. At 4-month follow-up, despite recovery in patient's neurological status, flexion deformity reappeared with recurrence of dropped head due to C7 pedicle screws pull-out. However, this was successfully managed with extension of the construct to the upper thoracic levels.

## 1. Introduction

Dropped head syndrome or head ptosis is a reducible flexion deformity of the neck that is caused from a weakness of the extensor muscles or increased tone of the flexor muscles of the neck resulting in the chin-on-chest deformity and at the extreme the patient will be unable to look straight ahead [1–6]. Notably, this flexion deformity is not fixed and can be corrected by extreme effort for a few minutes or by passive head extension and spontaneously by lying supine [1–6]. Heffner Jr. et al. were the first who defined dropped head syndrome in 1977 [7]. Later, it was highlighted that the syndrome can be seen in isolation or in association with a variety of generalized neuromuscular disorders as well as radiotherapy of the neck for corresponding malignancies [1–8].

The isolated type of dropped syndrome is a disease of the elderly that is caused by noninflammatory myopathy restricted to the paraspinal muscles of the neck being described by Suarez and Kelly Jr. for the first time in 1992 [9]. Subsequently, in 1996, the term isolated neck extensor myopathy (INEM) was proposed by Katz et al. [10].

Isolated dropped head syndrome proceeding cervical spondylotic myelopathy and their ultimate association is quite rare. This combination was first described by Kawaguchi in 2004 and since then only four more cases have been described in the literature [11–14].

Herein, a new case of cervical myelopathy developing two years after the appearance of dropped head syndrome, as a sequel of isolated neck extensor myopathy, is presented and a brief review of the literature on the condition is also provided [11–14].

## 2. Case Report

**Figure 1:**
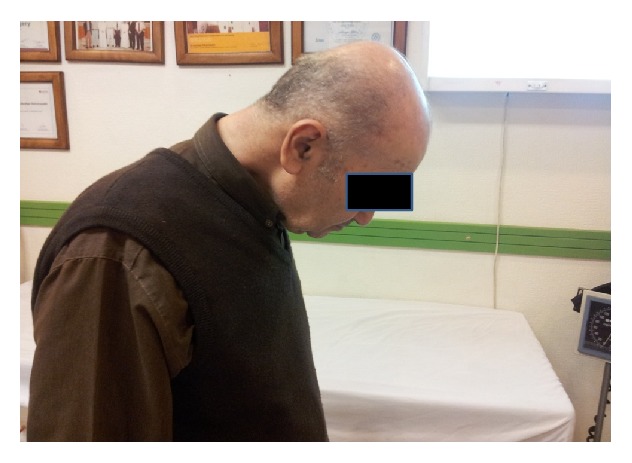
Photograph of the patient with dropped head syndrome in 2011.

**Figure 2:**
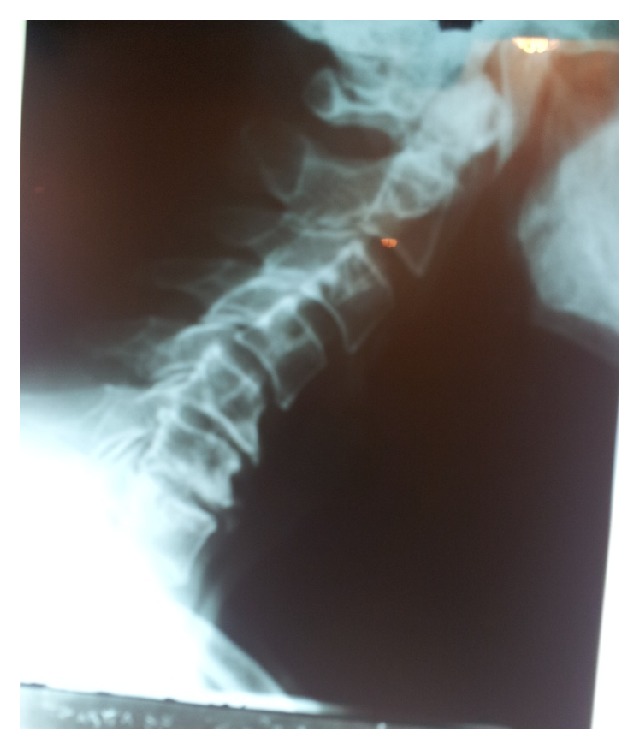
Lateral cervical spine in 2011 demonstrating mild subluxation of C4 with relation to C5 as well as degenerative changes at C5-C6 and C6-C7 levels.

**Figure 3:**
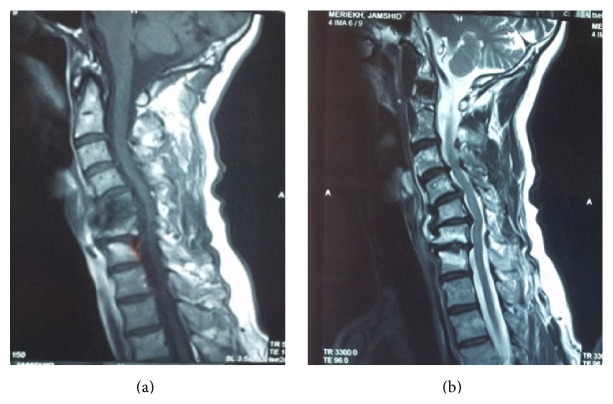
T1- and T2-weighted MRI and sagittal image in 2011 which had revealed cervical spondylosis with mild spinal cord compression.

**Figure 4:**
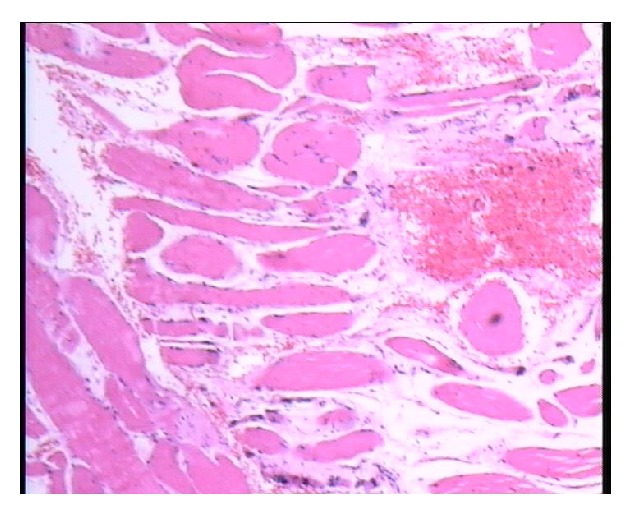
Muscle biopsy: a few muscle fibers are atrophic and the remaining have variable size.

This previously healthy 67-year-old man was admitted with chin to chest deformity in February 2011. The deformity had progressed rapidly from mild difficulty in keeping the head up to head drop over a period of 5 months (Figure 1). Upon admission, he could maintain his head up with extreme effort only for about five minutes. However, he was able to correct the deformity passively with his hands and the deformity could be relieved spontaneously in a supine position. He denied any other weakness in his extremities or difficulties in chewing and swallowing. The dropped head position had severely impaired the patient's activities of daily living and withdrawn him from social contacts. He used to wear a collar for outdoor activities such as shopping but he preferred to stay home most of the times. For most of his activities and for having a meal, he used to hold his head with the left hand instead of the collar. This type of correction was repeated several times in a day. Neurological examination showed normal parameters. Cervical spine radiographs revealed degenerative changes and flexion deformity of the neck (Figure 2). MRI revealed cervical spondylotic changes with mild compression of the spinal cord (Figures 3(a) and 3(b)). Clinical diagnosis of isolated neck extensor myopathy (INEM) was suspected. This was confirmed through neurophysiological evaluation with needle electromyography which revealed myopathic changes in the muscles of the neck and open biopsy of paravertebral muscles showing muscle fibers of variable size or atrophic compatible with myopathy (Figure 4).

Routine laboratory studies, such as serum creatine kinase (CK) and lactate dehydrogenase (LDH), were normal. Thyroid function tests, parathyroid hormone, acetylcholine receptor antibodies, and tumor markers were negative.

As he refused to undergo surgery, he was advised to wear a cervical collar to improve his neck posture and social interactions. However, according to his wife, he rarely wore the collar.

Over a period of two years and in particular during the last season, he exhibited mild but progressive weakness of all his extremities with difficulty in buttoning or unbuttoning his shirt and mild difficulty in walking owing to an unsteady gait. He also experienced tingling in both hands. These new difficulties along with the chin to chest deformity impaired his activities of daily living more than before and forced him to seek medical advice. This time, he could keep his head up for only one minute.

His neurological examination revealed spastic quadriparesis with positive Hoffman's sign, hyperactive reflexes, and equivocal extensor planter response in both sides.

**Figure 5:**
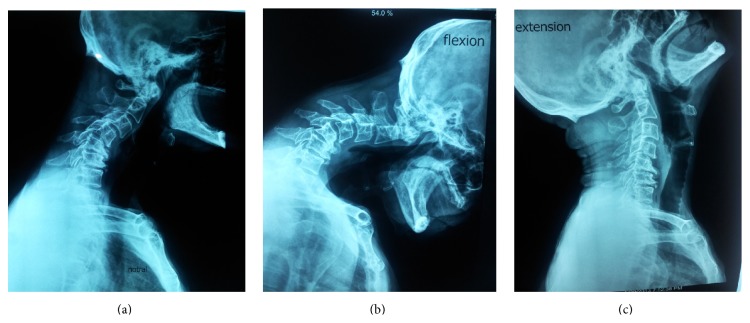
(a) Lateral cervical spine in neutral position in 2013, showing kyphotic spine with marked subluxation of C3 on C4 and C4 on C5 as well as degenerative changes at C5-C6 and C6-C7. (b) Lateral flexion radiograph. (c) Lateral cervical flexion and extension cervical radiographs indicating flexibility of the deformity.

**Figure 6:**
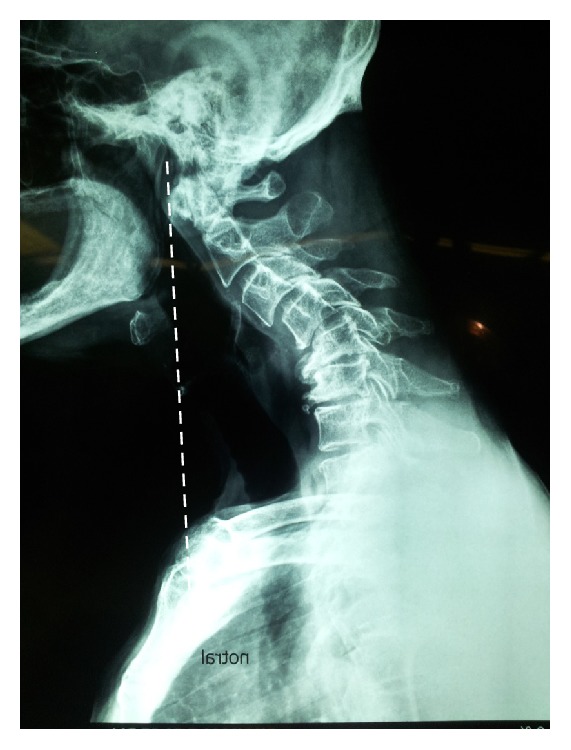
Note that the basion plumb line which stands posterior to manubrium.

**Figure 7:**
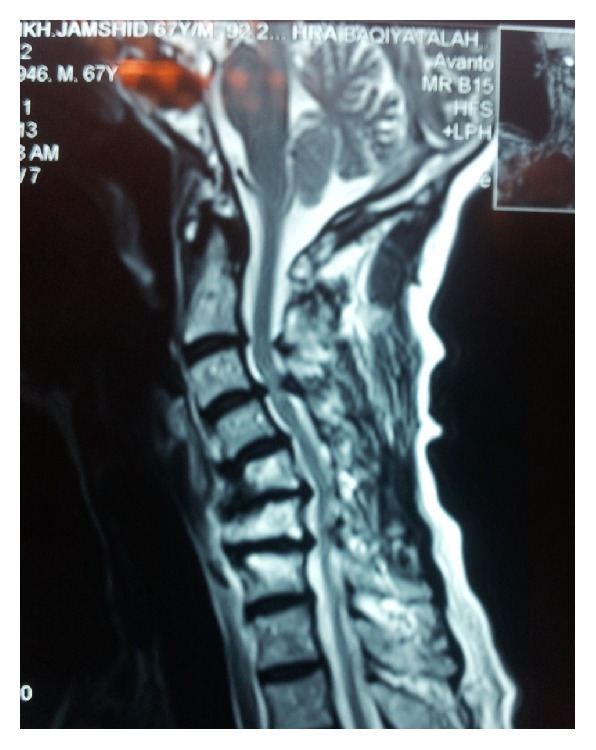
T2-weighted sagittal image in 2013, revealing aggravation spondylotic changes with moderate cord compression; note hyperintensity at C3-C4 level.

Cervical plain radiographs in dropped position disclosed osteoporotic cervical spine with severe kyphosis as well as instability with forward subluxation at C3-C4, C4-C5, and C5-C6 levels (Figure 5(a)). Flexion extension radiographs confirmed reducibility of the deformity (Figures 5(b) and 5(c)). In neutral position radiographs, the plumb line dropped from the basion to the posterior to the manubrium (Figure 6). New MRI, compared with the previous one which was taken in 2011, showed significant progression of spondylotic changes as well as myelopathic changes at C3-C4 level (Figure 7).

**Figure 8:**
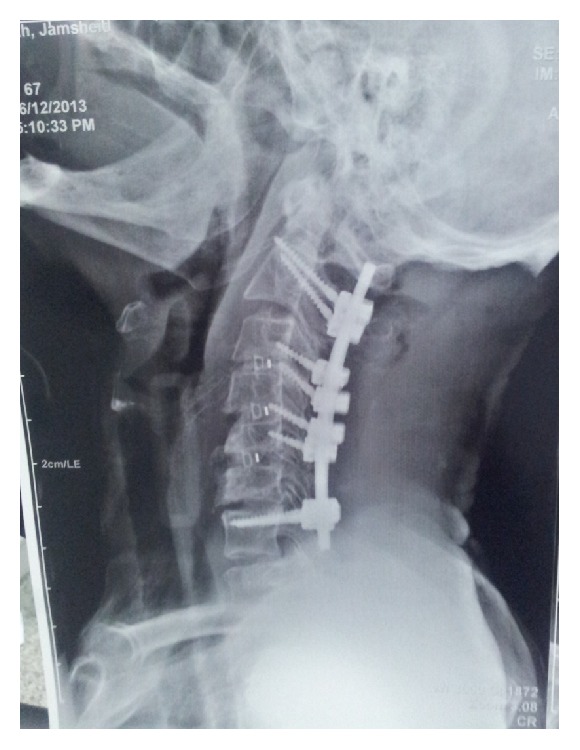
Postoperative lateral cervical radiograph, 3-level ACDF, and C2 to C7 pedicle screw resulting in optimal correction of the deformity.

One stage circumferential surgery was decided with respect to the osteoporosis. Therefore, a three-level anterior cervical discectomy fusion with cage at C3-C4, C4-C5, and C5-C6 was accomplished initially. Anterior procedure was followed with C3 to C6 laminectomy from C2 to C7 screw-rod stabilization. With the application of this strategy, simultaneous decompression of the spinal cord and correction of the deformity could be achieved. Postoperative course was uneventful and he was discharged in three days. Postoperative radiographs disclosed normal position of the neck (Figure 8). Two months after surgery, his neurological exam was nearly normal except for some brisk reflexes. He was satisfied and was thankful that surgery had significantly influenced his daily activities and interactions.

**Figure 9:**
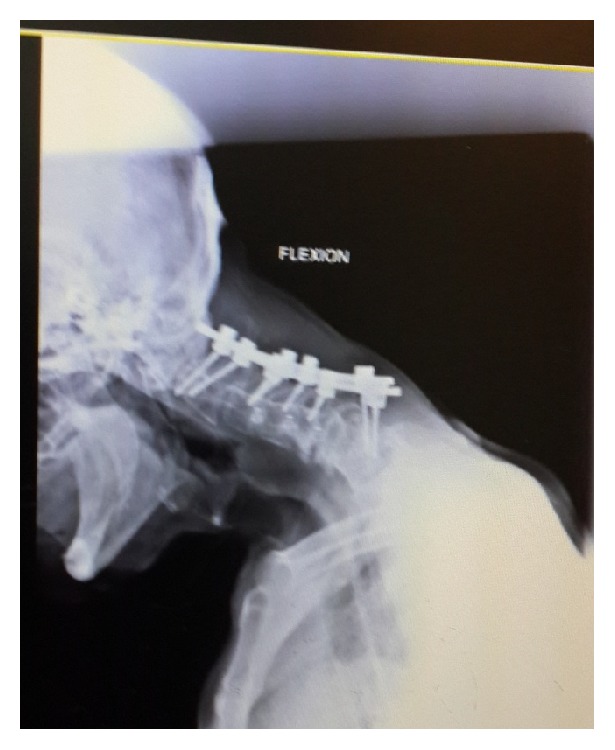
Failed instrumentation; note C7 pedicle screws which are pulled out.

But surprisingly, four months after surgery, his head has tendency to drop again, X-ray revealed recurrence of flexion deformity of the neck and out-pulling of both pedicle screws from the body of C7 (Figure 9). Redoing surgery in order to extend the construct to the upper thoracic vertebras was suggested which was accepted by the patient.

**Figure 10:**
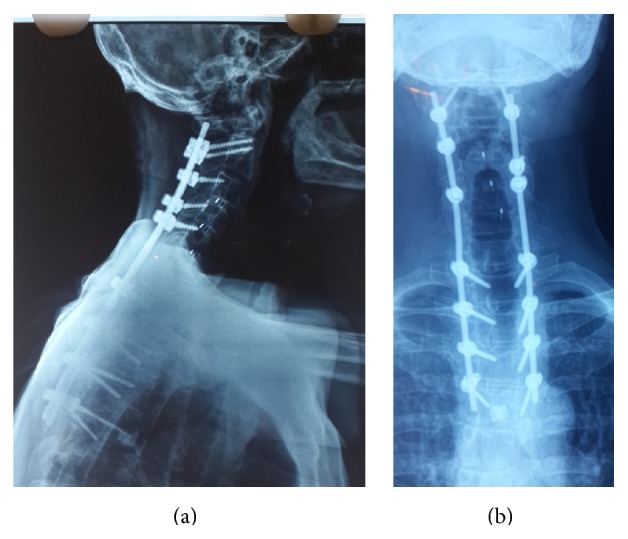
(a) Lateral cervicothoracic X-ray showing correction of the deformity 18 months after surgery. (b) Face cervical radiograph, indicating proper placement of the screws.

**Figure 11:**
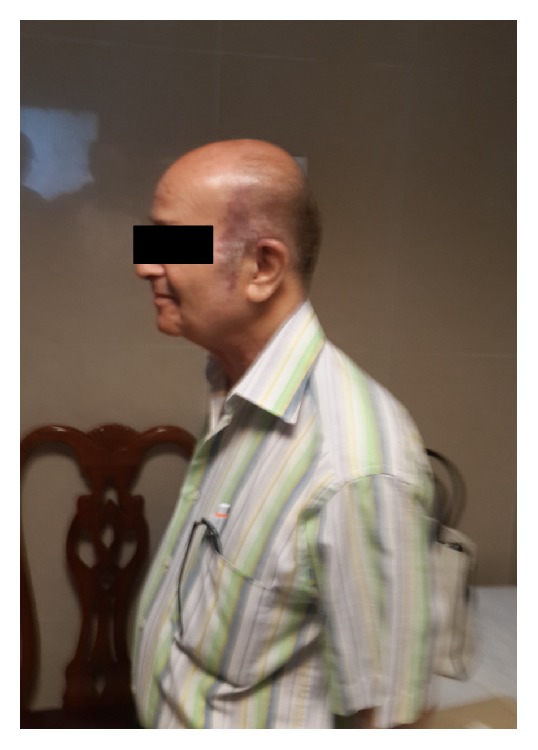
Photograph of the patient 18 months after surgery. Both the patient and the surgical team are satisfied.

With the patient in prone position the site of previous surgery was reopened and the rods and subsequently the screws of C7 were removed. Pedicle screws from T1 to T4 were inserted and the construct was extended from C2 to T4. Finally, the nuts were tightened with the head in normal position. Postoperatively, the patient was discharged after 3 days in Minerva collar, whereas the control radiographs were quite satisfactory (Figure 10). Now 18 months after revision surgery, the normal head and neck posture is preserved and he has a dramatic improvement in his quality of life, enabling him to perform the daily activities (Figure 11).

## 3. Discussion

Development of cervical spondylotic myopathy a few years after appearance of dropped head syndrome is a rare scenario. The information obtained from systematic review of the literature indicates that the since the report of the first example of this combination described by Kawaguchi et al. in 2004, four more cases have been published so far [11–14]. The information about the age, sex, type of surgery, and final outcome of these 6 patients including the current case is demonstrated (Table 1). According to this survey, the age of the affected patients was from 64 to 80 years with a mean of 70.83. Five out of six reported cases were females. Premyelopathy period for the dropped head syndrome varied from one to two years. The patients' symptoms were gradually relieved in all after cervical corrective surgery with instrumentation.

This association might be explained with two different theories. In the first theory, disturbances of spinal cord microcirculatory are regarded as the major factor. Accordingly, ischemia caused by cervical spondylosis results in preferential degeneration of the anterior horn cells of the cervical spinal cord. Eventually, this will result in weakness limited to the extensor muscles of neck causing dropped head syndrome. Later, with consideration of the natural course of cervical spondylosis, with further affection of the cord, the clinical picture of myelopathy will appear [11–13].

According to the second theory, with consideration of the age of the patients suffering from DHS due to INEM, association of asymptomatic cervical spondylosis with this syndrome should be quite frequent. Actually, as the head falls forward greater stress will be imposed on the neck extensors where restless efforts for correction of the kyphotic deformity combined with frequent failure of these efforts for holding the head up increase the work-load on the discoligamentous structures of the cervical spine. Gradually, this scenario can aggravate preexisting cervical spondylosis and, with progression of degenerative changes, cervical myelopathy will appear [11–13].

However, the rarity of this association remains a question, if we accept the fact that dropped head syndrome due to INEM is confined to the elderly and in this age group asymptomatic cervical spondylosis is not infrequent.

Nonetheless, coexistence of dropped head syndrome (DHS) and cervical spondylotic myelopathy (CSM) sooner or later will severely compromise the patient's quality of life and may result in significant disability if left untreated [11–14].

For achieving a good outcome and long life expectancy, appropriate surgical intervention for this association is required [11–14]. Conservative treatment is considered, in the patients with serious comorbidities, but is limited to strengthening exercises and wearing collars. Cervical collars, despite their ability to maintain the head in an upright position, are frequently not tolerated well by the patient, and they may lead to a pressure sores under the chin and on the occiput [2, 3, 13].

Surgery seems to be an obvious therapeutic option in association of DHS with cervical spondylotic myelopathy. However, owing to the paucity of information regarding surgical intervention, there is no clear consensus on the optimal approach or timing [11–14]. Nonetheless, it seems that after the establishment of the diagnosis and before significant implications on the health and quality of life occur, early surgery should be done. Once the clinical picture of cervical myelopathy and in particular quadriparesis appears, the possibility of rapid progression of myelopathy entailing in profound disability should be born in mind. If the dropped head is complicated with cervical spondylotic myelopathy, treatment of both conditions should be targeted [11–14]. This means that cervical cord decompressive surgery and correction of kyphosis are the mainstay of treatment in this combination, but the surgical approach should be individualized to the patient. To achieve these goals, an appropriate surgery can be performed with either a circumferential or posterior only approach. In fact, both combined anterior-posterior and posterior only decompression and stabilization have been advocated [2, 3, 15, 16].

Actually, the combination of DHS and CSM is a complex cervical spine pathology where the compressive effect of protruded multilevel degenerated intervertebral discs is aggravated by cervical kyphosis. This complex situation might benefit from circumferential surgery, if the protruded cervical disc causes canal compromise in particular at the site of myelopathy [13, 16]. Combination of anterior release with cervical discectomy and its replacement with standalone cages combined with laminectomy and posterior stabilization will guarantee lordotic posture and thorough cord decompression.

In fact, in DHS with kyphotic cervical deformity, once lordosis is not achieved with neck extension, disc release and reconstruction of the anterior column will facilitate correction and prevent failure which happens with gradual degeneration and subsequent collapse of the disc spaces that might occur with time. This progressive scenario might result in failure of posterior construct. Suboptimal correction has been demonstrated in some reported cases with DHS [15, 16].

Moreover, severe osteoporosis which coexists in the elderly with dropped head syndrome may complicate laminectomy plus posterior instrumentation. In osteoporotic subjects, strengthening of the anterior column with anterior discectomy and fusion might be helpful in prevention of posterior construct failure.

The distal length of posterior instrumentation was not clearly defined in the literature till recently that a formulation was proposed by Riew [3]. According to him, the extent of instrumentation and indication for incorporation of the thoracic spine in an ideal construct depend on the extent of kyphotic deformity and its severity based on the basion plum line [3]. Accordingly, if on lateral cervical spine a plum line dropping from the basion falls behind the manubrium, cervical instrumentation from C2 to C7 suffice [3]. But if the plum line falls anterior to the manubrium, cervicothoracic instrumentation will be required. However, as it was clearly demonstrated in the current case, this formulation did not work and despite the plumb line falling posterior to manubrium, C2 to C7 instrumentation was insufficient and our construction failed. Therefore, it seems that it is better to extend the construct to the upper thoracic spine in all the patients who suffer from dropped head syndrome in isolation or as a combined pathology [2, 3, 14, 17–19], in particular with consideration of the natural course of INEM which might be the progression of isolated myopathy to the muscles of the upper thoracic spine with time [18].

Whether the cranium should be included in the construct or not had been a matter of controversies in the past. In pre-screw-rod era, a contoured Steinman pin or a rod connecting the cranium to the cervical spine with the aid of the wires or hooks was the only choice for correction and stabilization of a dropped head [2]. This method was widely used and remained an accepted mode of surgery of kyphotic neck deformities for many years [12]. Even after introduction of cervical screws, extension of the construct to the skull was not stopped in DHS [20, 21]. According to proponents such method provides stronger construct rostrally, but it is at the cost of loss of rotation. However, extension to the cranium was gradually eliminated after description of C2-C1 transarticular screw and C2 pedicle screws [2, 3, 13, 14, 17–19]. Gerling and Bohlman in 2008 reported nine cases of DHS in the context of INEM that were managed with posterior instrumented fusion [18]. The surgical constructs for all patients spanned C2 to upper thoracic levels. This procedure has advantage of retaining some rotation upper cervical levels [2, 3, 13, 14, 18]. C2 pedicle screw can pull back the upper cervical spine till the desired curve is obtained [2, 3, 14, 17–19]. Even in osteoporotic patients, combining C2 pedicle screws in addition to an atlas hook on each side can provide a very strong encore for this purpose.

Information about the long-term outcome in combination of head drop and cervical spondylotic myelopathy, owing to its rarity in the literature, is limited. However, in this association, if the deformity is left untreated and decompression is not done, catastrophic results owing to the progression of myelopathy will ensue. In contrast to the dropped head syndrome secondary to serious neuromuscular diseases, which usually have a grave prognosis, in the combination of INEM and CSM, the outcome depends on the time of the surgery. If surgery is accomplished before the establishment of myelopathy, prognosis will be good, but with delay in diagnosis and from diagnosis to management the myelopathic changes may become irreversible with poor prognosis.

It should be noted that the dangers that are hidden in the correction of the fixed cervical kyphosis are not usually seen in this flexible kyphotic deformity. However, the patient should be informed about the restricted motions of the neck and the risk of increased falls because of an inability to see the walking surface.

In summary, progression of spondylotic changes with appearance of myelopathy should be expected in a patient with dropped head syndrome as a sequel of INEM. Periodic neurological examination every six months and control MRI in one-year interval seems justified. However, once DHS is complicated with early symptoms CSM, in the absence of serious comorbidities, in order to prevent disability, early surgery is indicated. Mainstay of surgery in this association is combination of decompression and instrumentation. Overall trend of instrumentation should be toward C2 to upper thoracic spine which provides lower-profile constructs with multiple points of fixation yielding stronger stabilization with an enhanced likelihood of successful fusion. However, extension of the construct to the cranium is not mandatory and usually not necessary.

## Figures and Tables

**Table 1:** Review of the cases with dropped head syndrome associated with cervical spondylotic myelopathy.

Author(s)	Year	Sex	Age	Type of surgery	Outcome
Kawaguchi et al. [11]	2004	F	80	Cervical laminoplasty from C2 to C6	Fair
Nakanishi et al. [12]	2007	F	68	C3-C4 laminectomy + occiput to T2 hook rod fixation	Good
Rahimizadeh and Afsari [13]	2013	F	72	C3–C6 laminectomy + C2–C7 pedicle screw-rod fixation	Good
Koda et al. [14]	2015	F	72	Laminectomy + C2–T4 screw-rod fixation	Good
Koda et al. [14]	2015	F	64	C4-C5 + C5-C6 ACDF + laminectomy C3 to C6 + C2 to T6 screw-rod fixation	Good
Present case	2016	M	69	C3-C4, C4-C5, C5-C6 ACDF + laminectomy C3 to C6 + C2–T4 screw-rod fixation	Good

## Abbreviations

DHS: Dropped head syndrome
CSM: Cervical spondylotic myelopathy.
